# Fever in pregnancy and the risk of congenital malformations: a cohort study

**DOI:** 10.1186/s12884-017-1585-0

**Published:** 2017-12-08

**Authors:** L. Sass, S. K. Urhoj, J. Kjærgaard, J. W. Dreier, K. Strandberg-Larsen, A.-M. Nybo Andersen

**Affiliations:** 1 Department of Public Health, University of Copenhagen, Øster Farimagsgade 5A, Box 2099, 1014 København K, Denmark; 2 Child and Adolescent Health Clinic, Juliane Marie Center, Research Unit for Women’s and Children’s Health, Rigshospitalet, Blegdamsvej 9, 2100 Copenhagen Ø, Denmark; 3 Department of Economics and Business Economics, Aarhus University, Fuglesangs Allé 4, DK-8210 Aarhus V, Denmark; 4 Ulsevej 43, 2650, Hvidovre, Denmark

**Keywords:** Pregnancy, Fever, Hyperthermia, Congenital malformations, Congenital anomalies

## Abstract

**Background:**

In a variety of animal species, hyperthermia in pregnancy has been recognized as teratogenic. Hyperthermia interferes with protein synthesis via heat-shock proteins, which can entail membrane disruption, cell death, vascular disruption, and placental infarction. This can induce severe fetal malformations or death. Fever during pregnancy, especially during embryogenesis, has also been associated with congenital malformations in human offspring.

The purpose of this large cohort study of clinically recognized pregnancies was to investigate whether fever during first trimester was associated with an increased risk of congenital malformations in the offspring.

**Methods:**

The Danish National Birth Cohort is a population-based cohort of 100,418 pregnant women and their offspring recruited in 1996 to 2002. Information on fever during pregnancy was collected prospectively by means of two telephone interviews.

The study population comprised the 77,344 pregnancies enrolled in the Danish National Birth Cohort where self-reported information on fever during first trimester of pregnancy was available. Pregnancy outcomes were identified through linkage with the National Patient Registry. Congenital malformations within the first three and a half years of life were categorized according to EUROCAT’s classification criteria. Logistic regression models were used to estimate the associations between fever in first trimester and overall congenital malformations and congenital malformations by subgroups.

**Results:**

Eight thousand three hundred twenty-one women reported fever during first trimester (10.8%) and 2876 infants were diagnosed with a congenital malformation (3.7%). Fever during first trimester did not affect the risk of overall fetal congenital malformation (OR 0.99, 95% CI 0.88–1.12). The subgroup analyses indicated slightly higher risk of congenital anomalies in the eye, ear, face and neck (OR 1.29, 95% CI 0.78–2.12) and in the genitals (OR 1.17, 95% CI 0.79–1.12), whereas lower risk of malformations in the nervous system (OR 0.47, 95% CI 0.21–1.08), the respiratory system (OR 0.56, 95% CI 0.23–1.29) and in the urinary subgroup (OR 0.58, 95% CI 0.35–0.99) was suggested, the latter constituting the only statistically significant finding.

**Conclusions:**

Overall, this study did not show any association between maternal fever in pregnancy and risk of congenital anomalies.

## Background

Congenital malformations are in the top 20 of leading causes of burden of disease globally [1] and are a significant contributor to infant mortality with 11,3% of neonatal deaths caused by congenital malformations [2]. Congenital malformations may result in long-term disability and thereby have significant impact on individuals, families, health care systems, and societies [3]. Malformations may have infectious, genetic or environmental origin but for the majority the etiology remains unknown [2].

For several decades, hyperthermia in pregnancy has been recognized as a teratogen in a variety of animal species including guinea pigs, rats, mice, pigs, sheep, and monkeys [4–6]. Hyperthermia interferes with protein synthesis via heat-shock proteins, which can entail membrane disruption, cell death, vascular disruption, and placental infarction [7–10]. All these mechanisms can induce severe fetal malformations or death.

Several case-control studies in human beings have associated fever in pregnancy with among other neural tube defects [11–14], cardiac malformations [15–18], oral clefts [19, 20], and renal malformations [21]. The nature of the specific malformations appears to depend on the extent, duration and timing of maternal fever [7], and it is therefore important to include information about the timing of these febrile episodes.

In this study, we investigate the association between fever in pregnancy and fetal congenital malformations in a large population-based cohort. We focused on fever in first trimester of pregnancy, when any teratogenic effect on the fetus is most plausible to occur. We expected more pronounced associations between fever and congenital anomalies the higher the temperature, the longer the duration of the febrile incident and with the time of fever during embryogenesis. Furthermore, we expected the association between timing of fever episodes and the different congenital malformation subgroups to vary due to the timing of the organogenesis.

## Methods

### Study population

Data was obtained from the Danish National Birth Cohort (DNBC), which is an ongoing population-based cohort of 100,418 pregnant women and their offspring recruited between March 1996 and November 2002 [22].

General practitioners invited pregnant women to participate in the study at their first antenatal care visit. To be able to participate in DNBC, the women had to: be pregnant, intend to carry their pregnancy to term, speak Danish well enough to be interviewed by telephone in Danish, and have a permanent address in Denmark. The women consented to be interviewed twice during pregnancy and allowed information concerning their own and their offspring’s health to be obtained from national registries.

Of all the enrolled pregnancies (*n* = 100,418), those that resulted in birth of live-born singletons (*n* = 92,670) and where the woman had participated in both the first (*n* = 86,783) and the second interview (*n* = 80,781) were included in the study. Mother–infant pairs with more than one week of discrepancy between the mother’s report of gestational age in the two interviews (*n* = 1567) were excluded as the information regarding timing of fever were considered unreliable. Likewise, all mother-infant pairs with incomplete information on fever (*n* = 919) were excluded**.**

Incomplete information about any of the a priori defined confounders: parity (0, 1, 2 or ≥3), previous spontaneous abortion (yes or no), maternal age at conception (≤24, 25–29, 30–34 and ≥35), in vitro fertilization (yes or no/irrelevant), maternal diabetes mellitus (yes or no) or rheumatoid arthritis (yes or no), medical treatment of epilepsy (yes or no), binge drinking, defined as drinking five or more drinks at one occasion, during first trimester of pregnancy (yes or no), smoking during pregnancy (yes or no), and socio-occupational status (higher grade professionals, lower grade professionals, skilled workers, unskilled workers, students, and unemployed >1 year) (*n* = 951) also resulted in an exclusion from the study. The remaining 77,344 mother–infant pairs were included in the analyses.

### Exposure

In this study the exposure of interest was fever during first trimester. Information about fever episodes was obtained in the two computer-assisted telephone interviews, where the first interview was completed at a median gestational age of 16 weeks (10th to 90th percentiles: 12–29 weeks) and the second at a median gestational age of 31 weeks (10th to 90th percentiles: 28–36 weeks). For the majority of women, information regarding fever was acquired from the first interview. For 7026 women, who participated in the first interview before the end of the 12th gestational week, we also used information about number and timing of fever episodes in the first trimester from the second interview. In both interviews, the women were asked about occurrence of fever, the maximum temperature, the timing of the fever episodes, the number of days with fever, and the number of fever episodes. English translations of the interviews are available at www.dnbc.dk.

In the analyses of the intensity of fever exposure, the maximum temperature was categorized as below 39 °C, over 39 °C, or unknown. The rationale for this categorization was based on literature showing that the teratogenic threshold effect in many species begins approximately at 1.5 °C above normal core body temperature [7].

Duration of fever was calculated as the longest period of consecutive days with fever (1 day, 2–3 days, 4 days or more, or unknown) and the fever episodes were divided into groups according to gestational age (pregnancy week 1–4, 5–8, and 9–12).

### Outcome

Denmark has a Civil Registration System where all citizens are registered with a unique 10-digit identification number used in all registries to identify the person. The identification numbers were used to link information from the Danish National Patient Registry, where pregnancy outcomes including information on congenital malformations were identified [23].

Congenital malformations in live-born children were diagnosed by physicians according to the International Classification of Disease 10th version (ICD-10), and all children diagnosed with ICD-codes Q00.0 to Q99.9 during the first three and a half year of life were identified.

The congenital malformations were divided into categories according to the classification criteria of the European Surveillance of Congenital Anomalies (EUROCAT) as seen in Table 3, where the ICD-10 codes are also specified [24]. Isolated minor congenital malformations (*n* = 1692) were excluded as cases. According to EUROCAT, minor congenital malformations are characterized as malformations with lesser medical, functional or cosmetic importance [24] .

A small group of infants with aneuploidy (*n* = 36) was not included as cases, as this chromosomal anomaly is a result of errors in mitosis or meiosis happening before conception.

Some of the congenital malformation categories were joined to form a broader group. The ear, face and neck subgroup was pooled with the eye subgroup and the musculo-skeletal- and limb categories were also united. Finally, in addition to the ICD-10 codes defined by EUROCAT, the category “Other” included the subgroups with abdominal malformations and the chromosomal malformations other than aneuploidies.

Children with more than one malformation were included in each appropriate anomaly subgroup, but were only counted once in the “Overall malformations” group.

### Covariates

Information on several covariates was obtained for main- and sub analyses (see Table 1). Information on the majority of the covariates was derived from the first interview, with the exception of medical treatment of epilepsy, use of antipyretics, and maternal infections, which was derived from the second interview.

**Table 1:** Characteristics of the 77,344 women with live-born singletons according to maternal fever in 1st trimester

	Total			Fever in 1st trimester		No fever in 1st trimester	
	N	%	PP^a^	N	%	N	%
Total study population	77,344	100	3.7	8321	100	69,023	100
Parity							
0	36,017	46.6	4.1	3375	40.6	32,642	47.3
1	28,843	37.3	3.4	3502	42.1	25,341	36.7
2	10,159	13.1	3.4	1191	14.3	8968	13.0
≥ 3	2325	3.0	3.2	253	3.0	2072	3.0
Previous spontaneous abortion							
No	62,570	80.9	3.7	6646	79.9	55,924	81.0
Yes	14,774	19.1	3.8	1675	20.1	13,099	19.0
Maternal age at conception							
≤ 24	9656	12.5	3.7	1012	12.2	8644	12.5
25–29	32,370	41.9	3.8	3477	41.8	28,893	41.9
30–34	26,466	34.2	3.7	2940	35.3	23,526	34.1
≥ 35	8852	11.4	3.6	892	10.7	7960	11.5
In Vitro Fertilization							
No/Irrelevant	75,646	97.8	3.7	8178	98.3	67,468	97.8
Yes	1698	2.2	4.7	143	1.7	1555	2.3
Maternal diabetes							
No	77,127	99.7	3.7	8285	99.6	68,842	99.7
Yes	217	0.3	6.5	36	0.4	181	0.3
Medical treatment of maternal epilepsy							
No	77,120	99.7	3.7	8306	99.8	68,814	99.7
Yes	224	0.3	9.4	15	0.2	209	0.3
Maternal rheumatoid arthritis							
No	77,243	99.9	3.7	8312	99.9	68,931	99.9
Yes	101	0.1	8.9	9	0.1	92	0.1
Binge drinking in 1st trimester							
No	59,006	76.3	3.7	6218	74.7	52,788	76.5
Yes	18,338	23.7	3.8	2103	25.3	16,235	23.5
Smoking during pregnancy							
No	57,719	74.6	3.7	6116	73.5	51,603	74.8
Yes	19,625	25.4	3.8	2205	26.5	17,420	25.2
Socio-occupational status							
Higher grade professionals	18,443	23.9	3.7	2118	25.5	16,325	23.7
Lower grade professionals	24,247	31.4	3.6	2716	32.6	21,531	31.2
Skilled workers	21,635	28.0	3.9	2127	25.6	19,508	28.3
Unskilled workers	10,697	13.8	3.6	1084	13.0	9613	13.9
Students	1782	2.3	4.3	217	2.6	1565	2.3
Unemployed >1 year	540	0.7	4.3	59	0.7	481	0.7
Child sex							
Male	39,546	51.1	4.0	4211	50.6	35,335	51.2
Female	37,798	48.9	3.5	4110	49.4	33,688	48.8
Pre-conceptional intake of folic acid							
No	67,892	87.8	3.7	7283	87.5	60,609	87.8
Yes	9353	12.1	3.8	1029	12.4	8324	12.1
Total	77,245	99.9	3.7	8312	99.9	68,933	99.9
Missing	99	0.1	3.0	9	0.1	90	0.1
Antipyretic use in 1st trimester							
No	65,205	84.3	3.8	6558	78.8	58,647	85.0
Yes	11,982	15.5	3.5	1748	21.0	10,234	14.8
Missing	157	0.2	4.5	15	0.2	142	0.2
Infections in 1st trimester							
No	64,164	83.0	3.7	6012	72.2	58,152	84.3
Yes	12,145	15.7	3.8	2196	26.4	9949	14.4
Missing	1035	1.3	3.2	113	1.4	922	1.3

The Danish National Birth Cohort 1996–2002

^a^PP: Prevalence Proportion of overall congenital malformations per 100 live births

Socio-occupational status was the household combined socio-occupational status, defined by the highest status within the couple. The information on maternal pre-diabetes mellitus was based on a combination of self-report and data from the National Patient Registry and this variable did not distinguish between Type I and Type II diabetes. Pre-conceptional intake of folic acid was self-reported at the time of recruitment to the cohort, and child sex came from the Civil Registration System. Maternal infections encompassed; urinary tract infections, vaginal infections, respiratory tract infections, gastroenteritis and childhood diseases.

### Statistical analysis

The associations between fever in the first trimester of pregnancy and 1) all congenital malformations and 2) congenital malformations by subgroups were estimated as odds ratios (OR) using logistic regression. The estimated ORs were presented with 95% confidence intervals (CI). Crude and adjusted analyses were performed and the a priori defined covariates were included in the adjusted analyses*.*

Some of the children in the cohort were born to the same mother and to take this into account we estimated 95% CI using robust standard errors.

### Sub analyses

To consider the influence of maternal infections during first trimester and use of antipyretics in first trimester on the estimated relationships, a first degree interaction between fever in first trimester, maternal infections and antipyretic use, respectively, was evaluated for overall congenital malformations using a Wald test.

Furthermore, the association between various measures of fever in first trimester (presented in Table 2) and overall congenital malformations was estimated while further adjusting for maternal infections in first trimester. Also, stratified analyses of the association between various measures of fever in first trimester and overall congenital malformations according to antipyretic use in the first trimester were performed.

**Table 2:** Risk (OR) of overall congenital malformations according to characteristics of fever in 1st trimester

	N	N Cases	Congenital Malformations OR Crude [95% CI]	Congenital Malformations Adjusted* OR [95% CI]
Fever				
No	69,023	2574	1 [Ref]	1 [Ref]
Yes	8321	302	0.97 [0.86–1.10]	0.99 [0.88 1.12]
Gestational age at fever incidents				
No fever	69,023	2574	1 [Ref]	1 [Ref]
Pregnancy week 1–4	1032	43	1.12 [0.82–1.52]	1.13 [0.83–1.54]
Pregnancy week 5–8	3407	124	0.98 [0.81–1.17]	0.99 [0.83–1.19]
Pregnancy week 9–12	3882	135	0.93 [0.78–1.11]	0.95 [0.79–1.13]
Maximum temperature				
No fever	69,023	2574	1 [Ref]	1 [Ref]
< 39.0 ° C	2613	108	1.11 [0.91–1.35]	1.12 [0.91–1.36]
≥ 39.0 ° C	2562	98	1.03 [0.84–1.26]	1.06 [0.86–1.30]
Unknown	3146	96	0.81 [0.66–1.00]	0.82 [0.67–1.01]
Duration of fever episode				
No fever	69,023	2574	1 [Ref]	1 [Ref]
1 day	1647	55	0.89 [0.68–1.17]	0.90 [0.69–1.18]
2–3 days	4799	180	1.01 [0.86–1.17]	1.02 [0.88–1.19]
+ 4 days	1873	67	0.96 [0.75–1.23]	0.98 [0.76–1.25]
Unknown	2	0	..	..

The Danish National Birth Cohort 1996–2002

*Adjusted for parity, previous spontaneous abortion, maternal age at conception, IVF, diabetes mellitus, medical treatment of maternal epilepsy, rheumatoid arthritis, binge drinking in first trimester, smoking during pregnancy, and socio-occupational status

## Results

In total 2876 children of 77,344 live-born singletons in this study were diagnosed with a congenital malformation before the age of three and a half years. The overall malformation prevalence in the study population was 3.7%, and this figure was higher for offspring of mothers with diabetes mellitus (PP = 6.5%), rheumatoid arthritis (PP = 8.9%), and offspring of women receiving medical treatment for epilepsy (PP = 9.4%),

Overall, 46.6% of the mothers were nulliparas while 37.3% had given birth once before. However, in the subgroup of women with fever in first trimester only 40.6% were nulliparas while 42.1% were primiparas, Table 1.

Overall, 8321 (10.8%) women reported at least one episode of fever within the first 12 weeks of pregnancy. Of the women reporting fever, only 62,2% reported the maximum temperature and of these almost half (49,5%) reported fever incidents with a temperature of 39 °C or more. In contrast, almost all women provided information about the duration of the fever incident and the distribution can be seen in Table 2.

The analyses revealed no increased risk of overall congenital malformations when the exposure was fever at any point during first trimester of pregnancy (OR 0.99, 95% CI 0.88–1.12). The risk of congenital malformations was slightly higher if the fever episode occurred during the 1–4 gestational week (OR 1.13, 95% CI 0.83–1.54), though not statistically significant. The estimates for the other gestational periods were not associated with increased risk of congenital malformations and the same accounts for the intensity of fever and the number of days with fever, Table 2.

### Sub analyses

Wald test showed no sign of interaction between fever in first trimester and maternal infections and antipyretic use, respectively (*p*-value = 0.95; p-value = 0.20). None of the estimates for the relationship between the various measures of fever in first trimester and overall congenital malformations as presented in Table 2 changed appreciably when further adjusting for maternal infections in first trimester or in the analyses stratified according to the use of antipyretics in first trimester (results not shown).

In relation to the subgroups of congenital malformations, fever during first trimester was associated with slightly increased risk of congenital malformations in the eye, ear, face and neck (OR 1.29, 95% CI 0.78–2.12) and in the genitals (OR 1.17, 95% CI 0.79–1.71), however, none of them statistically significant. Furthermore, a decreased risk of congenital malformations was seen in the following categories: nervous system (OR 0.47, 95% CI 0.21–1.08), urinary tract (OR 0.58, 95% CI 0.35–0.99) and respiratory system (OR 0.56, 95% CI 0.23–1.29). Of these, only the decreased risk of urinary tract congenital malformations was statistically significant. The remaining subgroups of congenital malformations were not associated with exposure to fever during first trimester of pregnancy, Table 3.

**Table 3:** Risk (OR) of specific congenital malformation if the mother had fever during 1st trimester of pregnancy

	N Cases	CRUDE OR Fever in 1st Trimester OR [95% CI]	ADJUSTED^a^ OR Fever in 1st Trimester OR [95% CI]
Type of Congenital Malformation			
Overall Malformations^b^	2876	0.97 [0.86–1.10]	0.99 [0.88–1.12]
Nervous system (DQ00 – DQ07)	114	0.46 [0.20–1.05]	0.47 [0.21–1.08]
Eye, ear, face and neck (DQ10 – DQ18)	134	1.29 [0.78–2.12]	1.29 [0.79–2.10]
Congenital heart defects (DQ20 – DQ26)	656	0.99 [0.77–1.27]	1.01 [0.79–1.29]
Respiratory system (DQ30 – DQ34)	79	0.56 [0.23–1.39]	0.56 [0.23–1.39]
Orofacial clefts (DQ35 – DQ37)	163	0.90 [0.54–1.51]	0.90 [0.54–1.50]
Digestive system (DQ38 – DQ45, DQ790)	198	0.98 [0.63–1.55]	1.00 [0.63–1.56]
Genital (DQ50 – DQ52,DQ54 – DQ56)	249	1.14 [0.78–1.67]	1.17 [0.79–1.71]
Urinary (DQ60 – DQ64,DQ794)	231	0.58 [0.34–0.97]	0.58 [0.35–0.99]
Limb and musculoskeletal (DQ650-DQ652,DQ58-DQ660, DQ681-DQ682, DQ688, DQ69 – DQ75, DQ761-DQ764, DQ766-DQ769, DQ77-DQ78, DQ796, DQ798-DQ799)	1157	1.04 [0.87–1.25]	1.06 [0.88–1.28]
Other (DQ27-DQ28, DQ80-DQ85, DQ87, DQ89, DQ93, DQ99, DQ792, DQ793,DQ795)	297	1.00 [0.69–1.45]	1.01 [0.70–1.46]

Classification of malformations with ICD-10 codes derived from EUROCAT

The Danish National Birth Cohort 1996–2002

^a^Adjusted for parity, spontaneous abortion, maternal age at conception, IVF, diabetes Mellitus, medical treatment of maternal epilepsy, rheumatoid arthritis, binge drinking in first trimester, smoking during pregnancy and socio-occupational status

^b^Cases with more than one malformation are only counted once in the “Overall malformations” subgroup

## Discussion

In this large cohort study, fever during first trimester of pregnancy was not significantly associated with congenital malformations diagnosed within the child’s first three and a half years of life. The analyses demonstrated that there are no statistically significant association between fever in first trimester and any of the congenital malformation subgroups, except for a decreased risk of malformations in the urinary system. The reduced risk, although statistically insignificant, of having a congenital malformation in the nervous system is unexpected considering results from previous studies where central nervous system defects are among the most common consequences of hyperthermia [7, 11, 13, 25–29]. Since most malformations in the nervous system are extremely severe, a possible explanation might be that many of the fetuses with malformations in the nervous system were aborted before recognition of pregnancy or before the first antenatal care visit at the general practitioner and thereby before a possible enrollment into the DNBC.

A previous study reported that fever in the first 16 weeks of pregnancy did not increase the risk of spontaneous abortions in clinically recognized pregnancies in this cohort [8], and thus it seems unlikely that these congenital malformations resulted in spontaneous abortions at a later state in pregnancy.

During the study period, women over 35 years and women at high risk of having a child with congenital malformations were offered prenatal screening [22]. Hence, another feasible explanation of the decreased risk of having a child with malformations in the nervous system could be that the women, who had an ultrasonic examination diagnosing the fetus with severe malformations in the nervous system, subsequently had an induced abortion. Since the study population only includes live-born singletons, it is not possible to rule out that induced abortions could help explain the decreased risk of malformations in the nervous system. In order to address this question, we estimated the risk of induced abortion due to fetal disease among the women in the DNBC who participated in the first interview after week 12 (*n* = 82,003). The analysis indicated an increased risk of termination of pregnancy due to fetal abnormality (OR 1.56, 95% CI 0.96–2.55) if the women had fever during first trimester of pregnancy. Hence, this might partly explain the unexpected finding regarding nervous system anomalies.

The identification of a statistically significant reduced risk of giving birth to a child with urinary congenital malformations after having fever during first trimester of pregnancy found in this study does not correspond to the findings in a previous study [21]. However, as a number of statistical tests were conducted on the same overall study population this may be a chance finding and thus the result of a statistical type 1 error.

Previous studies on maternal fever and congenital malformations have reported different results. This inconsistency may be caused by differences in one or more study factors including the method used for measuring body temperature, duration and timing of fever episodes, recall bias in retrospective studies, small study sizes, and confounding.

Generally, participation rates in large cohort studies have diminished over the last decades to around the level observed in DNBC, where approximately 60% of the invited pregnant women in Denmark participated [22]. Because of the relatively low participation rate in the DNBC, selection bias is a possibility. The enrolled women have previously been observed to be slightly healthier overall than women in the source population; nevertheless, this have been shown to have very limited impact on risk estimates in DNBC [30], and the complete register-based follow-up of the cohort furthermore protects against selection bias.

Nonetheless, selection may still be the reason why a marginally lower proportion of overall congenital malformations is seen in our cohort when compared to the background population during the same time period [31]. In our cohort, the proportion of children diagnosed with a congenital malformation (including minor malformations) was 6% compared to 6.5% in the background population.

Higher socioeconomic profile and healthier life style are presumed to explain these differences, as women enrolled in cohorts have been shown to be healthier and more well-off [31–34]. Thus cohorts are selected samples, but baseline-selection is by definition independent of outcome and thereby does not introduce selection bias in the exposure-outcome association. However, baseline-selection may impact the confounding pattern, thus it is different from the source population of each cohort [35]. Strengths of the present study include the large sample size, the prospectively collected information on fever, the detailed information on body temperature, duration and timing of fever episodes, the nearly complete follow-up and the adjustment for a variety of covariates. Furthermore, since all confidence intervals in this study are relatively narrow the accuracy of the estimate is high.

A limitation in this study was that, like most studies on birth anomalies, only live-born children were included, since congenital malformations in stillbirths, spontaneous abortions and medically terminated pregnancies are not registered in Denmark.

Additionally, the relatively limited number of children with congenital anomalies in this study did not allow us to stratify the outcome further than the overall groups of congenital malformations recommended by EUROCAT. This lumping of specific malformations into body system groups might have masked slightly increased risk of the rarest malformations.

Information on fever in first trimester was collected prospectively, i.e. before pregnancy outcome was known, and thus any misclassification of exposure was non-differential, leading to an underestimation of the association. The lacking information on how body temperature was measured imparts an uncertainty to the data regarding temperature. Nonetheless, body temperature was (in the times of the data collection) usually measured with a rectal thermometer and the temperature information indicated can therefore likely be regarded as a measure of core temperature [8]. Additionally, the maximum temperature is not necessarily the correct maximum temperature for the event but the highest value measured.

The overall validity of data on diagnoses from the National Patient Registry is considered to be acceptable for general surveillance and epidemiological research [35], also when specifically considering congenital malformations [32]. Children diagnosed with a congenital malformation until the age of three and a half years were included in the study, and this period ensured that the majority of congenital malformations usually not detected at birth were also included. However, uncertainty and inaccuracies in the diagnoses are still seen in national registries, but incorrect diagnoses can at most lead to non-differential misclassification, which would result in an underestimation of the association.

## Conclusions

While most case-control studies indicate positive associations between maternal fever and selected congenital anomalies, the results of this large-scale cohort study do not support a relation between maternal fever during first trimester of pregnancy and congenital anomalies.
